# Increase in protandry over time in a long‐distance migratory bird

**DOI:** 10.1002/ece3.9037

**Published:** 2022-07-06

**Authors:** Johanna Hedlund, Thord Fransson, Cecilia Kullberg, Jan‐Olov Persson, Sven Jakobsson

**Affiliations:** ^1^ Centre for Ecology and Conservation, College of Life and Environmental Sciences University of Exeter Penryn UK; ^2^ Lund University Lund Sweden; ^3^ Department of Environmental Research and Monitoring Swedish Museum of Natural History Stockholm Sweden; ^4^ Department of Zoology Stockholm University Stockholm Sweden; ^5^ Department of Mathematics Stockholm University Stockholm Sweden

**Keywords:** bird migration, climate change, phenology, *Phylloscopus*, protandry, willow warbler

## Abstract

Protandry is a widespread life‐history phenomenon describing how males precede females at the site or state of reproduction. In migratory birds, protandry has an important influence on individual fitness, the migratory syndrome, and phenological response to climate change. Despite its significance, accurate analyses on the dynamics of protandry using data sets collected at the breeding site, are lacking. Basing our study on records collected during two time periods, 1979 to 1988 and 2006 to 2016, we aim to investigate protandry dynamics over 38 years in a breeding population of willow warblers (*Phylloscopus trochilus*). Change in the timing of arrival was analyzed in males and females, and protandry (number of days between male and female arrival) was investigated both at population level and within breeding pairs. Our results show advancement in the arrival time at the breeding site in both sexes, but male arrival has advanced to a greater extent, leading to an increase in protandry both at the population level and within breeding pairs. We did not observe any change in sex ratio that could explain the protandry increase, but pronounced temperature change has occurred and been reported in the breeding area and along the migratory route. Typically, natural selection opposes too early arrival in males, but given warmer springs, this counteracting force may be relaxing, enabling an increase in protandry. We discuss whether our results suggest that climate change has induced sex‐specific effects, if these could be evolutionary and whether the timing of important life‐history stages such as arrival at the breeding site may change at different rates in males and females following environmental shifts.

## Graphical abstract

Protandry is a widespread phenomenon in nature, influential on several aspects of life history in migratory birds. Despite its significance, accurate analyses are lacking on its dynamics. Here, using a unique data set spanning 38 years, we show how protandry has increased over time during a period of temperature warming, suggesting that climate change may have cause sex‐specific effects in the willow warbler.

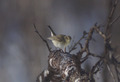

## 1 INTRODUCTION

Animal migration is a widespread natural phenomenon, common in a vast diversity of species groups, from mammals and reptiles to birds and insects. In the pursuit of seasonal resource peaks, or to escape unfavorable local conditions, billions of migrants travel, sometimes thousands of kilometers across oceans and continents, in order to reach another destination. In the European‐African flyway alone, 2.1 billion migratory birds fly between their breeding areas and wintering grounds (Hahn et al., 2009). The survival and reproductive success of individuals is dependent on accurate timing, that is, the phenology of migration (Alerstam et al., 2003; Newton, 2007; Visser & Gienapp, 2019), and the mechanisms that govern bird migration can be complex and flexible. For example, earlier breeding is related to increased fitness in birds (Svensson, 1997; Verhulst & Nilsson, 2008), but may also entail higher costs, since the risk of adverse conditions is greater earlier in the year (Newton, 2007). Within species, arrival to, and departure from, the breeding site can differ between sex and age of the birds (Ahola et al., 2004; Both & te Marvelde, 2007; Gill et al., 2013; Gordo, 2007; Hedlund et al., 2015), suggesting divergent benefits and costs to individual characteristics.

When male birds time their arrival at the breeding ground to proceed the arrival of conspecific females, the phenomenon is known as protandry, meaning simply “males first.” Protandry is common, in migratory passerines (Coppack & Pulido, 2009; Rubolini et al., 2004), in other bird groups (Gordo et al., 2013; Huyvaert et al., 2006; Newton, 2007) and in plants, insects, amphibians, mammals, and fish (Forrest, 2014; Morbey & Ydenberg, 2001). There are several, nonmutually exclusive hypotheses aimed at explaining the phenomenon of protandry in birds (Coppack & Pulido, 2009; Kokko et al., 2006; Morbey & Ydenberg, 2001). Among these, the “the mate opportunity hypothesis” has received most attention. This states that, since male fitness relies more strongly on number of matings than female fitness (Andersson, 1994; Ball & Ketterson, 2008), males risk reduced fitness (i.e., reduced mating opportunities) if they arrive at the breeding ground around the same time as females (Kokko et al., 2006; Wiklund & Fagerström, 1982). Thus, there is a direct selective advantage for males to arrive earlier (Velmala et al., 2015), as supported by studies demonstrating that early arriving males have more opportunities for extra‐pair matings (Canal et al., 2012; Cooper et al., 2011; Reudink et al., 2009; but see Tomotani et al., 2017), and a higher likelihood of being polygynous (Alatalo et al., 1984; Reudink et al., 2009).

In the majority of migratory birds where protandry is observed, it may appear to be continuously present as a phenomenon and species may be considered as protandrous, that is, the earlier arrival of males is taken as a constant and a fact for certain species. However, when efforts have been devoted to detailed and longer term studies of sex‐specific arrival, a few intriguing exceptions have been identified. In a study of two species of skuas with overlapping breeding areas (*Stercorarius maccormicki, S. antarctica*), male inter‐species cross‐breeding skewed the sex ratio to female‐biased, resulting in an otherwise protandrous species becoming protogynyous, that is, females arriving before males (note that when these skua species hybridize, it is always a pair consisting of a male of species S. maccormicki and a female of species S antarctica, resulting in a female‐biased operational sex ratio in S maccormicki) (Lisovski et al., 2016). In an investigation into protandry in barn swallows (*Hirundo rustica*), it was suggested that climate change had relaxed natural selection against too early arrival, enabling the sex under greater sexual selection to be early at the breeding ground (i.e., males) to advance arrival more, resulting in an increase in protandry (Møller, 2004). Lastly, in a long‐term monitoring effort of a population of white storks (*Ciconia ciconia*), a delay in male arrival was observed, resulting in a decrease in protandry. The cause for the decrease was suggested to be the parallel relaxation in selection for too early arrival (Gordo et al., 2013).

Sex ratio, the factor found to empirically influence protandry/protogyny in skuas, has also been identified theoretically as an important parameter in determining mating opportunities. Specifically, when modeled, a male‐biased sex ratio was found to be an even stronger determinant of protandry than extra‐pair matings, as not all males in a male‐biased population can gain mating opportunities with a social partner (Kokko et al., 2006). Thus, a change in sex ratio is presumed to be influential in the expression of protandry/protogyny.

Addressing the effect of climate change on protandry, it has been suggested that warmer springs could elicit a greater advancement of male than female arrival (Møller, 2004; Spottiswoode et al., 2006; Tøttrup & Thorup, 2008). As the intensity at which protandry is expressed is presumed to be generated under the opposing forces of natural and sexual selection, any fitness benefits associated with earlier arrival for males are balanced against viability costs (Morbey et al., 2012; Spottiswoode et al., 2006). For example, protandrous males may arrive at the breeding ground before a naturally selected optimum, with respect to temperature and resource availability (Brown & Brown, 2000; Irons et al., 2017). Due to climate change, early spring temperatures have increased (Høgda et al., 2013; Karlsen et al., 2007; The IPCC, 2013), leading to an earlier onset of the growing season (Høgda et al., 2013; Park et al., 2008) and earlier emergence of insects (Parmesan, 2006; Roy & Sparks, 2000; Visser et al., 2006). Thus, climate change has relaxed the constraints acting to oppose early spring arrival of migratory birds (Visser et al., 2015). Given the assumed higher selective pressure on males to arrive earlier compared to females, a resulting prediction is an increase in the degree of protandry due to climate change (Møller, 2004; Spottiswoode et al., 2006). Comparably, if there is a diminishing selection pressure for early male arrival, as seen in the example on white storks where reproductive success was not associated with first arrival (Gordo et al., 2013), the driver for protandry is diminished, with a decrease seen in the degree of protandry expressed.

There are strong indications that individuals, populations, and species differ in their ability to respond to climate change, with great inter‐ and intraspecific differences in phenological change reported in many bird systems (Both & te Marvelde, 2007; Gordo, 2007; Hedlund et al., 2015). Sexual selection has been put forward as one explanation behind the intraspecific variation (Gordo, 2007; Møller, 2004; Spottiswoode et al., 2006), with the hypothesis that differing selective pressures on males and females result in differing responses to phenological shifts. However, in spite of the prevalence of protandry in birds, and its suggested influence on individual fitness, population viability, migratory dynamics, and phenological response to environmental change (Rainio et al., 2007), there is a lack of analyses that have examined its dynamics over time. To date, the majority of studies have utilized records of passing conspecific males and females at bird observatories (Bauböck et al., 2012; Harnos et al., 2014; Rainio et al., 2007; Tøttrup & Thorup, 2008). By extrapolating the degree of protandry from bird observatory data, there is a risk of sampling a mixture of individuals from different breeding populations, thus comparing arrival days of males and females that do not breed in the same area and that are not essentially protandrous (Briedis et al., 2019; Coppack & Pulido, 2009; Maggini & Bairlein, 2012).

Here, we investigate protandry dynamics in a Swedish population of willow warblers (*Phylloscopus trochilus*) using a long‐term data set of arrival time of breeding pairs collected at the breeding site, consisting of two periods: 1979 to 1988 and 2006 to 2016 spanning 38 years. We analyze male and female arrival at the breeding ground during the study period, and test for possible changes in the degree of protandry and whether the sex ratio of the population has changed.

## 2 MATERIALS AND METHODS

### 2.1 Study species

**FIGURE 1:**
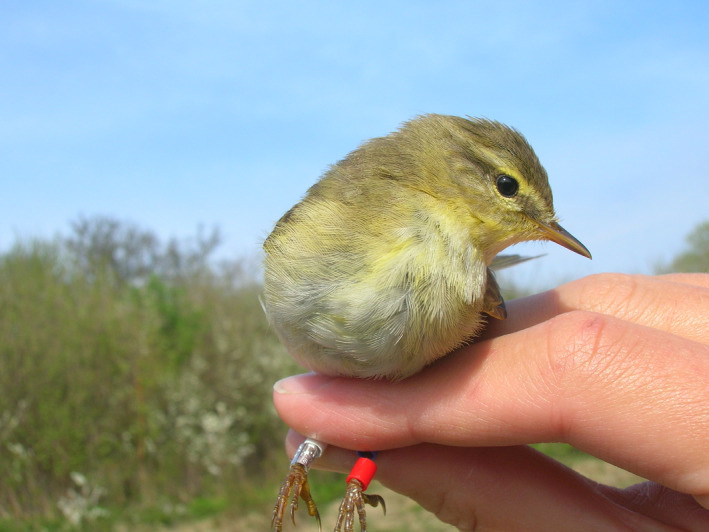
Willow warbler male with metal ring and color band. Photo: Johanna Hedlund

The willow warbler (Figure 1) is a small insectivorous long‐distance migrant that breeds in northern Eurasia and winters in sub‐Saharan Africa (Bairlein, 2006; Hedenström & Petterson, 1987). It is sexually dimorphic, with the male being larger (Norman, 1983). The species is very common in northern Europe and Russia, and is the most abundant migrant in the Eurasian‐African flyway, with 150 million birds estimated to migrate annually between the continents (Hahn et al., 2009). In Sweden, it is the most common bird species, with an approximated 13.2 million breeding pairs (Ottoson et al., 2012). However, the willow warbler has seen declines in both Sweden (Green & Lindström, 2020; Lehikoinen et al., 2014), and in Europe (EBCC, 2019) presenting a need to better understand the basic biology of the species.

### 2.2 Data collection

Field data were collected at Tovetorp Research Station (58°94′N, 17°14′E) in south‐central Sweden, from late April throughout May during the years 1979–1988, 2006–2009, and 2013–2016. Between 12 and 33 territories (mean = 21.3 ± SE 1.34/year) were monitored each year and visited daily. Willow warbler males establish territories in late April to early May in our study area (Hedlund et al., 2015; Jakobsson, 1988) and arrival dates for individual males was determined as the first day of territorial song activity (Jakobsson, 1988). Female arrival date was determined as the first day a female was observed paired with a territorial male as her social partner. Even though it is difficult, although not impossible, to distinguish the sex of a willow warbler in the field, the arrival of a female to a territory is apparent through distinct changes in song patterns and behavior of the territorial male (Radesäter et al., 1987). In addition, females were observed to initiate nest‐building soon after pair‐formation (Radesäter et al., 1987). All territories were monitored daily before male arrival, and beyond egg‐laying, ensuring an exact record of arrival dates of both sexes. Unbanded males were rarely seen in the study area suggesting low occurrence of nonterritorial males.

Willow warblers are often socially monogamous, but polygamy (Jakobsson, 1987) and extra‐pair matings occur (Bjørnstad & Lifjeld, 1996; Fridolfsson et al., 1997; Gil et al., 2007). Females may sometimes choose to pair with an already mated male, and in our study population so called “secondary females” were occasionally observed, but were excluded from the analysis of female arrival as the timing of their pair‐formation with the male was difficult to determine. All males were caught with mistnets and color‐banded (Figure 1), and many returned to breed in consecutive years (Hedlund et al., 2017; Jakobsson, 1988). In a few rare cases, individual males were too wary to be captured and remained unmarked. Unlike males, females were not regularly color‐marked, and are less philopatric in this species (Hedlund et al., 2017).

The degree of protandry was measured both within breeding pairs (defined as the difference in days between male and female arrival within a mated pair) and at population level (defined as the mean difference in days of arrival between all territorial males and all breeding females at the breeding site per year). A total of 309 records of protandry, including 398 individual arrival dates of males, and 309 individual arrival dates of females were included in the analysis.

Data for the degree of protandry and arrival dates were separated into two time periods for the purpose of the analysis, given that they are separated by almost twenty years. The first time period included all years between 1979 and 1988 and the second time period included 2006–2016, excluding the three years 2010, 2011, and 2012 (from here on we will not specifically mention that data is lacking for these 3 years).

We measured sex ratio as the proportion of all territorial males that remained without a social partner each year, so‐called unpaired males. As the vast majority of young are a result of within‐pair copulations in willow warblers in our study population (Gyllensten et al., 1990), acquiring a social partner is important for reproductive success in males, and an informative descriptor of operational sex ratio. It is our strong conviction that there are no, or extremely few, nonbreeding females in our population. Survival is very low between years in this species, and individual females are likely to have only one year to reproduce. Willow warblers breed in young forests, edge zones and early succession production forests, all common and plentiful habitats in the study area, therefore it is unlikely that territory availability is a limiting factor for reproduction; in accordance, we rarely observed nonterritorial males in the area.

**FIGURE 2:**
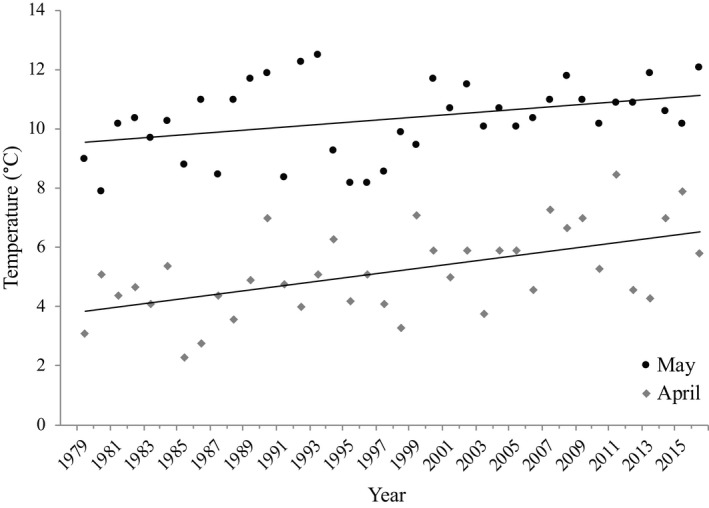
Increase in average monthly local spring temperatures in April (*r* ^2^ = 0.31; *p* < .001) and may (*r* ^2^ = 0.14; *p* < .02) during the study period (1979–2016)

During the study period, the local area experienced a pronounced increase in average spring temperatures in April and May (SMHI, 2022; see example data: Figure 2). However, we do not include an analysis of a climatic parameter's effects on arrival time and degree of protandry, as the complexity at which such a climatic model should be executed (see for example Haest et al., 2020) lies beyond the scope of this study. First, there is a difficulty in pinpointing appropriate localities at which a climatic parameter should be obtained (Gordo, 2007; Haest et al., 2020). A migrating bird is exposed to environmental conditions across a wide range of geographical areas, and a shift in migratory timing can be a response to environmental change at any one or a combination of these areas, for example, the overwintering site, stop‐over sites and/or the breeding site (Both, 2010; Both et al., 2005; Haest et al., 2020; Thingstad et al., 2015), and carry‐over effects could also be of importance (Marra et al., 1998). In addition, as degree of protandry is retrieved from both male and female arrival data, and as the sexes migrate at different times, there is also temporal ambiguity in the choice of climatic parameter. There is strong evidence of climate change increasing spring temperatures at the breeding site (Høgda et al., 2013; IPCC, 2013; Kivinen et al., 2017; SMHI, 2020). However, recent analysis suggests that the best variables in explaining willow warbler migratory phenology are temperature in continental Europe and wind patterns over the Mediterranean, rather than breeding site conditions (Haest et al., 2020).

### 2.3 Data analysis

We compared male and female arrival date and the degree of protandry between the first period of data collection (years 1979–1988) and the second period of data collection (years 2006–2016) using two mixed models, with year as a random factor, and period as a fixed factor. Male and female arrival, along with within‐population protandry, were analyzed in one mixed model and within‐pair protandry was analyzed in a separate mixed model. To avoid pseudo‐sampling males, as some males returned to breed at the same site in consecutive years, male ID was included in the analysis of male arrival and protandry (within pairs and at population level) as an additional crossed random factor. The models were fitted in Stata, version 15 (StataCorp, 2017). A mixed model was chosen since sample size differed between years, and because true yearly means within periods can differ, which argues against using yearly averages or pooling all observations within the two periods.

A potential change in the proportion of unpaired males between the two periods was investigated using Pearson's chi‐squared test, and here the number of unpaired males, and total number of males per year, were pooled for each period.

## 3 RESULTS

### 3.1 Arrival date of males and females

**FIGURE 3:**
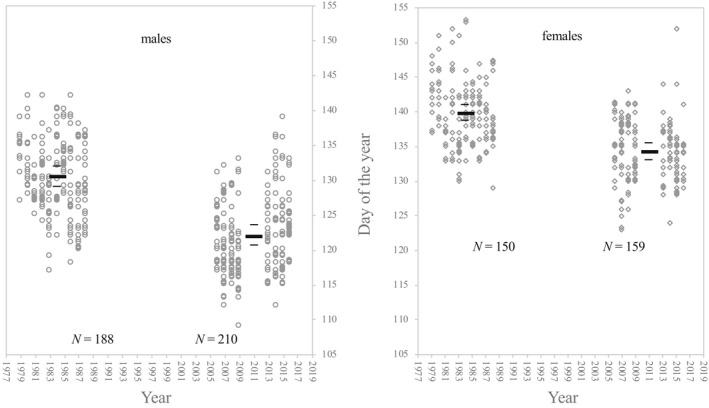
Arrival date of males (circles) to the left and females (diamonds) to the right for the two study periods, 1979–1988 and 2006–2016. Note that several individuals may arrive on the same date, and to illustrate this, the circles/diamonds have been slightly jittered around those dates affected. The vertical axes indicate day of the year, where day 105 corresponds to the date April 15th and day 155 to June 4th. Bars show mean arrival date with a 95% confidence interval for each sex for each period

We find that both male and female mean arrival date advanced significantly (*p* < .0005) from the first period (years 1979–1988) to the second period (years 2006–2016) (Figure 3). The variance accounted for by random effects was 11.4% for year and 17.2% for male ID. For males, mean arrival date advanced by 8.60 ± 1.91 days, from the day of the year 130 ± 1.31 (fixed effects estimate, 95% confidence interval) in the first period, to the day of the year 121 ± 1.39 (fixed effects estimate, 95% confidence interval) in the second period, which correspond to dates May 10th and May 1st, respectively. For females, the mean arrival date advanced by 5.50 ± 1.96 days, from day of the year 139 ± 1.34 (fixed effects estimate, 95% confidence interval) in the first period to day of the year 134 ± 1.43 (fixed effects estimate, 95% confidence interval) in the second period, which correspond to the dates May 19th and May 14th, respectively. The full model outcome is available in an Appendix A (Table A1).

### 3.2 Protandry

**FIGURE 4:**
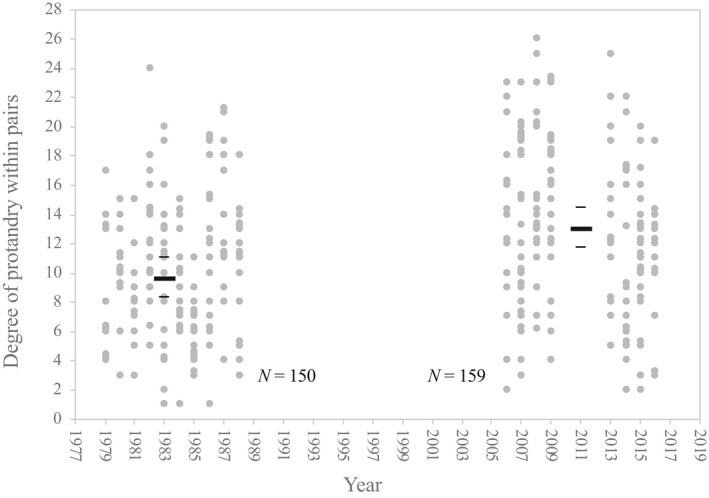
Degree of protandry for the two time periods considered (1979–1988 and 2006–2016), specified as the difference in arrival time (number of days), between males and females in a mated pair. Note that for several pairs, the degree of protandry may be the same in a given year and to illustrate this, the markers have been slightly jittered around those values. Bars show the mean degree of protandry for each period and 95% confidence intervals

Analysis of the mean degree of protandry within pairs was based on 150 breeding pairs in the first period, and 159 breeding pairs in the second period, and showed a significant increase over time (*p* < .0005). Specifically, from a mean of 9.92 ± 1.26 days in the first period (fixed effects estimate, 95% confidence interval), protandry increased by 3.14 days (±1.83) to a mean of 13.06 ± 1.26 days in the second period (fixed effects estimate, 95% confidence interval, Figure 4). Thus, there has been a considerable increase in the degree of protandry over the last to 30 years. The variance accounted for by random effects was 8.4% for year and 7.7% for male ID. Protandry at the population level showed very similar estimates, with the male population arriving 9.31 ± 1.07 days before the female population in the first period (fixed effects estimate, 95% confidence interval) and significantly increasing the difference in arrival (*p* < .0005) by approximately 3.11 (±1.48) days, arriving 12.43 ± 1.02 days before females in the second period (fixed effects estimate, 95% confidence interval). The variance accounted for by random effects was 11.4% for year and 17.2% for male ID. The full model outcomes are available in an Appendix A (Tables A1 and A2).

### 3.3 Sex ratio

The mean proportion of unpaired males for the first period was 20.21% (*N*_unpaired_ = 38, *N*_tot_ = 188) and 24.28% (*N*_unpaired_ = 55, *N*_tot_ = 210) for the second period. No difference between the periods was detected (*p* = .33).

### 3.4 Local temperature

In order to visualize potential local climate change, average monthly temperatures for April and May were acquired from the Swedish Meteorological and Hydrological Institute's open data portal “Luftwebb” (SMHI, 2022), for a location 30 km east (Oxelösund, 58°40′14.05″N; 17°06′5.47″E) of the study site. Temporal change in temperature across the years 1979 to 2016 was analyzed using linear regression, and an increased over time, in both April (*r* ^2^ = 0.31; *p* < .001) and May (*r* ^2^ = 0.14; *p* < .02), was detected (Figure 2).

## 4 DISCUSSION

We analyzed the temporal dynamics of protandry in a migratory bird using a high‐resolution long‐term dataset, focusing on a time period spanning 38 years. Specifically, we documented the arrival dates of male and female willow warblers in a breeding population during two periods, 1979–1988 and 2006–2016. Our results show that both males and females have advanced arrival to the breeding ground, with males now arriving 8.6 days earlier, and females arriving just over 5 days earlier than before. We also demonstrate a change in protandry over time, both at the population level, and within breeding pairs. The effect is strongest within individual breeding pairs, with males arriving on average 13 days before their social partner in the second period, in comparison to 9.9 days in the first period.

Confirmation of increases in protandry have previously been sought with limited success. In recently analyzed records of breeding pairs of pied flycatchers (*Ficedula hypoleuca*), no change in the degree of protandry was detected (Cadahía et al., 2017). Likewise, most analyses based on ringing records of passing migrant birds have not found any indications of increased protandry over time (Bauböck et al., 2012; Rainio et al., 2007; Tøttrup & Thorup, 2008), but see (Harnos et al., 2014). Importantly, protandry is a phenomenon most appropriately studied at the breeding site and bird observatory data is not suitable for the analysis. An increase in protandry based on a breeding site data set has been reported once before, in barn swallows (*Hirundo rustica*), during a 33‐year long study (Møller, 2004). The methodology of this study generates certain ambiguity, and it is the only other example, alongside ours, that has shown a temporal increase in protandry in a migratory bird at its breeding site. Unlike the barn swallow study, our data set is based on continuous, daily monitoring of individual male and female arrival to the breeding site, with individual males color‐banded every year, resulting in precise measurements of the degree of protandry within the population, as well as within breeding pairs.

In the literature, three factors have been previously identified as affecting the expression of protandry: sex ratio (Kokko et al., 2006), environmental change (Møller, 2004), and selection on too early arrival (Gordo et al., 2013). Sex ratio is suggested to determine whether all males can gain a social breeding partner. If they are unable to gain a social breeding partner, females are regarded as a scarce resource with theory predicting strong protandry (Kokko et al., 2006). In our study population, we detected no significant change in the percentage of un‐paired males over time, that is, social pairing did not appear to become more or less difficult for males, and we deem it very doubtful that a pronounced change in sex ratio occurred in the population during the study period. Thus, it appears unlikely that a sex ratio change is the cause behind the prominent increase in protandry in our results. In addition, the empirical knowledge on whether mating opportunities are lower for later arriving males in breeding populations with male‐biased sex ratio is limited (Samplonius & Both, 2017), and the few studies reporting on a change in protandry have not included sex ratio dynamics in their analyses (Gordo et al., 2013; Harnos et al., 2014; Møller, 2004). One study that did test the effect of more pronounced male‐biased sex ratio on protandry found no effect (Saino et al., 2010), but the results were based on species recorded at a bird observatory and not on breeding populations. Although a dramatic skewing of the sex ratio can have the power to switch a population from protandry to protogyny, as shown in the previously described unique example of skuas (Lisovski et al., 2016), and a male‐biased sex ratio is theoretically instrumental for protandry to appear and to be sustained (Kokko et al., 2006), there is no empirical evidence available to direct our prediction of how much sex ratio has to change to increase protandry in our system.

During the study period, the region in which the breeding area is located experienced a pronounced increase in spring temperatures (Figure 1; IPCC, 2013; Høgda et al., 2013; Kivinen et al., 2017; SMHI, 2020), indicating that birds arriving earlier in the second period would not see as cold temperatures as they would have if they arrived at the same date in the first period. Further south in continental Europe, a spatiotemporal analysis of climate effects on willow warbler migration has indicated that temperature change along the migratory route may have advanced arrival with 3 days (Haest et al., 2020). According to the hypothesis stating that a relaxing of natural selection against too early arrival would enable the sex under greatest sexual selection (males) to advance arrival (Møller, 2004; Spottiswoode et al., 2006; Tøttrup & Thorup, 2008), a rise in early spring temperatures could lead to an increase in protandry. We deem it likely that this is, at least to a great extent, an explanation to the pronounced increase in protandry documented at our study site.

Our data set does not contain enough information on reproductive success to make a temporal analysis into selection pressure on time of arrival possible. However, an analysis of the data from the earlier period at our study site show that arrival date is the best predictor of pairing date in male willow warblers, with earlier arrival being strongly associated with early pairing (Radesäter et al., 1987). This association has also been verified in later studies of willow warblers at other sites, both in Sweden and in Britain (Arvidsson & Neergaard, 1991; Gil & Slater, 2000). It is also evident that the timing of reproduction has become earlier in Swedish willow warblers, as egg‐laying date has advanced in pace with earlier arrival over time (Hedlund et al., 2015). Early breeding in migratory passerines is associated with higher fitness (Svensson, 1997; Verhulst & Nilsson, 2008), and as nest predation is high in willow warblers (Bjørnstad & Lifjeld, 1996), early breeding also increases the opportunity for re‐nesting attempts (Thingstad et al., 2015; Verhulst & Nilsson, 2008). Thus, early arrival and breeding should be advantageous for male willow warblers, and if it was not, we probably would not see an increase in protandry (Gordo et al., 2013). In conclusion, there is ample evidence that natural selection against too early arrival has relaxed, as early spring conditions throughout Europe have become milder, and there is supportive data showing that there should be a selection pressure on males to arrive early in willow warblers.

Interestingly, our result revealed an advancement in arrival for both sexes. In the few other studies where protandry increase has been observed (Harnos et al., 2014; Møller, 2004), temporal change in protandry was due to males being the only sex that advanced arrival over time. Similarly to males, females gain fitness benefits from early arrival by acquisition of high quality mates and territories and increased reproductive success (Bensch & Hasselquist, 1991; Kokko et al., 2006; Smith & Moore, 2005). Thus, females should also be under selection for earlier arrival (Kokko et al., 2006). The reason why females do not advance arrival in pace with males could be a combination of the stronger sexual selection on males, coupled with additional sex‐differentiated selection pressures. For example, females are the sole investors in egg‐laying and incubation, and as they lay their own body weight in eggs (Tiainen, 1983), their physical condition at the start of breeding is a crucial parameter influencing their reproductive success. The single most important factor determining breeding success in willow warblers is nest predation (Tiainen, 1983), and as females build their nest on the ground, they rely heavily on vegetation camouflage, making it suboptimal to arrive before the vegetation offers concealment from visual hunting predators. Arriving too early, in harsher conditions, may thus be more detrimental to females, especially since they are the smaller sex (Norman, 1983; Radesäter et al., 1987). In conclusion, even though both sexes may gain certain benefits from early arrival, it is likely to be associated with greater costs for females (i.e., the susceptibility hypothesis: Morbey & Ydenberg, 2001).

Whether migratory responses to climate change, such as advanced arrival dates, are driven by microevolutionary processes or are governed by phenotypic plasticity, is the subject of intense discussion (Bowers et al., 2016; Charmantier et al., 2008; Haest et al., 2020; Przybylo et al., 2000; Tarka et al., 2015; Visser et al., 2015). An endogenous basis for protandry has been demonstrated in laboratory studies, where it has been shown that males exhibit spring migratory activity (“zugunruhe”) before females in response to photoperiod cues (Coppack & Pulido, 2009; Terrill & Berthold, 1990; Widmer, 1999) and even under constant photoperiod and environmental conditions (Maggini & Bairlein, 2012). In addition, light‐level geolocators have shown that males initiate spring migration before females and that this is the reason for their earlier arrival at breeding grounds, giving support for the existence of a sex‐differential selection pressure operating on spring migration (Briedis et al., 2019; Pedersen et al., 2019; Schmaljohann et al., 2016). Recently, candidate genes for sex‐specific timing of migration were identified in the willow warbler (Bazzi et al., 2017). The authors of the study identified sex‐specific phenotypic effects at two loci, arguing that differing selection pressure on the timing of life‐history events in males and females, such as those producing protandry, may be the cause (Bazzi et al., 2017). Thus, there is support for protandry being an innate behavior with a genetic basis in willow warblers. However, whether a temporal increase in the degree of protandry, as found in our study, suggest a sex‐specific microevolutionary response to a warmer spring have to remain speculative, given the need to first show that a selective pressure has acted on the arrival dates. Indeed, variation in a complex trait such as reproductive timing may be the result of an interaction of drivers, including microevolutionary adaptation, but also nonadaptive environmental effects and carry‐over effects such as individual condition (Pulido, 2007), or sexually differentiated plastic responses (Harnos et al., 2014; Morbey et al., 2012).

Our study is unique in finding an increase in protandry using both population level and within‐pair measurements of differences in arrival, and consequently there is very little comparative, detailed knowledge on how protandrous behavior alters in nature. Whether the observed degree of protandry we report on has plateaued, if it will continue to increase, or if females will “catch up” with males leading to future decreases in protandry, are important outcomes to track in the future. Appreciating the interacting effects of climate change and sexually biased behaviors will be imperative to understand organisms' adaptive potential during current rapid global climate change.

## AUTHOR CONTRIBUTIONS

**Johanna Hedlund:** Conceptualization (equal); data curation (equal); formal analysis (equal); investigation (equal); methodology (equal); writing – original draft (lead); writing – review and editing (lead). **Thord Fransson:** Conceptualization (supporting); formal analysis (supporting); methodology (supporting); writing – original draft (supporting); writing – review and editing (supporting). **Cecilia Kullberg:** Conceptualization (equal); formal analysis (equal); investigation (supporting); methodology (supporting); visualization (supporting); writing – original draft (supporting); writing – review and editing (lead). **Jan‐Olov Persson:** Formal analysis (equal). **Sven Jakobsson:** Conceptualization (equal); data curation (equal); formal analysis (equal); funding acquisition (lead); investigation (equal); methodology (equal); visualization (equal); writing – original draft (supporting); writing – review and editing (supporting).

## CONFLICT OF INTEREST

No conflict of interest to disclose.

## Data Availability

The data supporting the study are available at the Dryad Digital Repository https://doi.org/10.5061/dryad.s1rn8pkbd.

## APPENDIX A

**TABLE A1:** Full model outcomes for the analysis of male and female arrival, and within‐population protandry

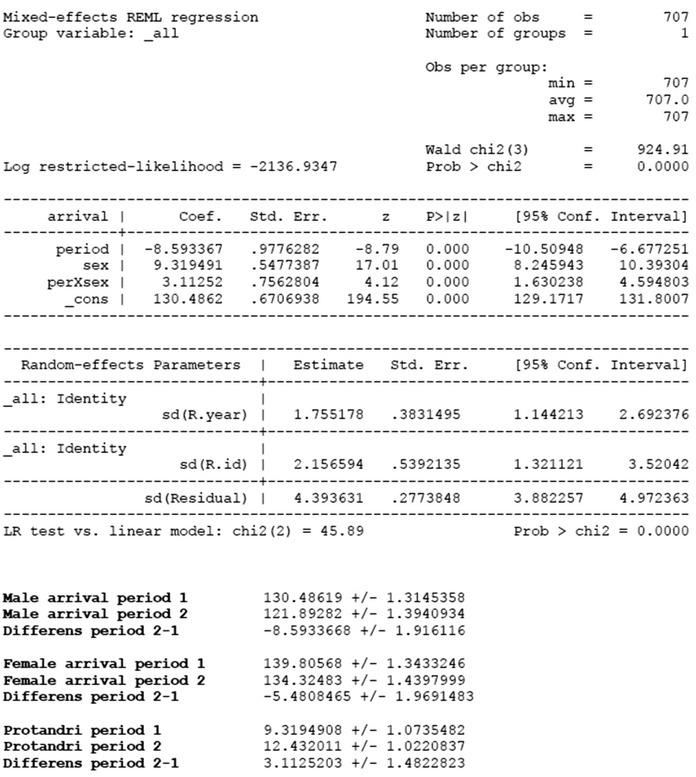

**TABLE A2:** Full model outcomes for the analysis of within‐pair protandry
